# Annual Disease Experience by Type and Correlations with Unmet Healthcare Needs among ROK Military Personnel

**DOI:** 10.1093/milmed/usz458

**Published:** 2020-05-18

**Authors:** Hwi Jun Kim, Sarah So Yeon Oh, Dong Woo Choi, Sun Yeong Won, Hae Jung Kim, Sung Chan Ko, Sung-In Jang, Eun-Cheol Park

**Affiliations:** 1 Department of Public Health, Graduate School, Yonsei University, 50 Yonsei-ro, Seodaemun-gu, Seoul 03722, Republic of Korea; 2 Institute of Health Services Research, Yonsei University, 50 Yonsei-ro, Seodaemun-gu, Seoul 03722, Republic of Korea; 3 Army Cadet Military School, Training & Doctrine Command, Republic of Korea Army, 177 Munmu-ro, Goesan, Republic of Korea; 4 Department of Logistic’s Management, Ministry of National Defense, 22 Itaewon-ro, Yongsan-gu, Seoul, Republic of Korea; 5 Department of Medical, Second Operational Command, Republic of Korea Army, Muyeol-ro, Suseong-gu, Daegu, Republic of Korea; 6 Armed Forces Medical School, Ministry of National Defense, 78-501 Jaun-ro, Yuseong-gu, Daejeon, Republic of Korea; 7 Department of Preventive Medicine, Yonsei University College of Medicine, 50 Yonsei-ro, Seodaemun-gu, Seoul, Republic of Korea

## Abstract

**Introduction:**

The National Statistical Yearbook of Defense 2018 issued by the Republic of Korea (ROK) Ministry of National Defense reported that the number of patients using military hospitals steadily increased from 2008 to 2017. However, in the outpatient clinic statistics for years 2015–2017 from the ROK Armed Forces Medical Command, the amount of medical care received from some medical departments, such as the infection medicine, surgery, and anesthesiology departments, decreased. Therefore, the purpose of this study was to observe the differences in incidence of military personnel’s unmet healthcare needs according to number of diseases by type.

**Materials and Methods:**

The study used data from the Military Health Survey, which was conducted from 2014 to 2015 and included 5162 responses from ROK military personnel. The number of diseases by type and unmet healthcare needs were self-reported. A multiple logistic regression analysis was used to examine the validity of the annual disease experience by type and correlations with unmet healthcare needs.

**Results:**

Of the 5162 military personnel, 25.2% experienced unmet healthcare needs, and the more people with the number of disease by type, the more likely they were to experience unmet healthcare needs (1: 13.4%, 2: 22.9%, 3: 29.2%, 4: 34.5%, 5: 41.4%). The logistic regression analysis also revealed significant differences (1 = REF, 2 odds ratio (OR) = 1.83, 95% confidence interval (CI): 1.50–2.24; 3 OR = 2.53, 95% CI: 2.05–3.11, 4 OR = 3.10, 95% CI = 2.49–3.85; ≥5 OR = 3.85, 95% CI = 3.08–4.81). In addition, subgroup analysis showed that female military personnel are more likely to experience unmet healthcare needs than are male military personnel. We have also confirmed that working areas and private insurance can affect unmet healthcare needs.

**Conclusion:**

This study suggests that unmet healthcare needs are influenced by the number of disease by the type of ROK military personnel. It is therefore necessary to strive to reduce the number of military personnel who experience unmet healthcare needs through this data.

## Introduction

Unmet healthcare needs are a result of the limited use of medical services, which can be attributed to the lack of financial, physical, and social access to medical services.^1–3^ Previous studies have found that factors such as minority factors, disease factors, and health behaviors are influential in the use of health services.^4^ There are also many demographic and social factors related to unmet healthcare needs; for example, gender, cost of services, lack of time attributable to work, and lack of medical facilities. Such unmet healthcare needs can affect disease prognosis in terms of early detection, increased severity, and increased likelihood of complications.^5^ Therefore, unmet healthcare needs are used as important indicators for measuring the accessibility of medical services worldwide.^6–10^

According to the Korean Constitution, the ROK (Republic of Korea) military exists for the sovereignty, national interest, and security of the people. It is an organization capable of lawfully exercising force.^11^ Therefore, the ROK Army, Navy, and Air Force are always conducting intensive training during peace time for ROK military operations.^12^ Thus, military personnel are constantly exposed to various diseases, such as musculoskeletal and respiratory diseases, because of intensive training and group life.^13–20^ Therefore, in order to ensure medical treatment whenever military personnel are sick, laws such as the law on military healthcare and the basic act on the status and service of military personnel have been put into practice. These stipulate the guarantee of military personnel’s medical rights.^21^^,^^22^ However, military personnel’s healthcare accessibility still needs to be improved due to the geographical characteristics of many troops in Gangwon and Gyeonggi provinces near the military demarcation line, as well as the occupational environmental characteristics that require approval from superiors to visit medical institutions.

The National Statistical Yearbook of Defense 2018 issued by the ROK Ministry of National Defense (MND) reported that the number of patients using military hospitals steadily increased from 2008 to 2017 (1 206 504 in 2008, and 1 606 206 in 2017). However, in the outpatient clinic statistics for years 2015–2017 from the ROK Armed Forces Medical Command (ROK AFMC), the amount of medical care received from some medical departments, such as the infection medicine, surgery, and anesthesiology departments, decreased. We thought this phenomenon would have an impact on the receipt of medical services according to military personnel awareness concerning type-specific diseases (Supplementary Table 1). In addition, in a study of the changes and trends in unmet healthcare needs of Koreans, following four items were analyzed: the Korea National Health and Nutrition Examination Survey (KNHANES), the Community Health Survey (CHS), the Korea Health Panel (KHP), and the Korea Welfare Panel Study. The number of Koreans who experienced unmet healthcare needs was 8.4–13.6%. On the other hand, about 2–3 times as many people experienced unmet healthcare needs in this study conducted on military personnel.^23^

In the case of Korea, representative secondary data concerning public health status are investigating unmet healthcare needs, but there is little data concerning the unmet healthcare needs experienced by military personnel. In addition, there has been a lack of prior research on unmet healthcare needs for military personnel worldwide. Most previous studies focused on the racial, mental health, and mortality aspects of military personnel and their families.^24–28^

Therefore, the present study analyzed the correlation between unmet healthcare needs according to the number of diseases by type in the ROK military. The aim of this study was to contribute to the development of future military healthcare policy.

## Methods

### Study participants

We obtained data for our study population from the 2014–2015 Military Health Survey (MHS), a cross-sectional, military sample survey conducted by the School of Military Medicine, which was designed to obtain military health determinants.

The MHS was developed based on the KNHANES to determine the health status of ROK military personnel. The KNHANES is an ongoing surveillance system in the ROK that assesses the health and nutritional status of Koreans, monitors trends in health risk factors and the prevalence of major chronic diseases, and provides data for the development and evaluation of health policies and programs in Korea.^29^ Similarly, the MHS collects data on military personnel’s general characteristics, health behaviors, injuries, safety consciousness, social support, disease and medical service use, and characteristics that only apply to female soldiers. To represent the entire population of ROK military personnel, the MHS utilizes stratified random sampling. Specifically, the MHS sampling considers soldiers’ sex, rank, service type, and working area.

Raw and secondary data were obtained from the Korean Military Medical School with the Dean’s approval. The institutional review board of the ROK AFMC provided formal ethical approval for the use of the MHS data sets (IRB approval number AFMC-14-IRB-004, AFMC-15060-IRB-15-049). Since the 2014 MHS is an anonymous, self-administered survey, individual responses cannot be linked to specific personnel or medical records. The total number of survey participants during the 2 years the study was conducted was 14 244 (officers and warrant officers: 1914, non-commissioned officers [NCO]: 3604, enlisted soldiers: 8726). We extracted responses from those who had experienced disease within the past year and those who reported having unmet healthcare needs experience. Furthermore, we only included cases that had responses to questions regarding sex, marital status, educational level, military type, branch, rank, working area, service classification, working type, working time, subjective health status, physical activity, smoking, private insurance, and satisfaction with military medical service. Finally, 5162 cases (officers and warrant officers: 866, NCO: 1726, enlisted soldiers: 2570) were included in the sample.

### Variables

Unmet healthcare needs experience within the past year is this study’s main dependent variable. In the MHS, the answers to this question were as follows: “I have experienced unmet healthcare needs,” “I have never experienced unmet healthcare needs,” and “I have never needed medical care or examinations at a medical clinic.” We conducted the study after excluding missing data from persons who answered that they did not need medical care or examinations.

The variable of interest was the number of diseases that individuals had within the past year. The MHS investigated “all diseases in the recent year” from the disease and medical use category. The categories included problems with eyes and ears (decreased vision, cataracts, glaucoma, amotio retinae, difficulty hearing, otitis media, perforation of the eardrum, and dizziness); the respiratory system (rhinitis, paranasal sinusitis, sinusitis, bronchiectasis, bronchitis, pneumonia, and cold); the immune system (asthma, atopic disease, allergic conjunctivitis, and allergic rhinitis); the cardiovascular system (acute myocardial infarction, angina pectoris, cardiac hypertrophy, arrhythmia, hypertension, hyperlipidemia, coronary atherosclerosis, cerebral infarction, cerebral hemorrhage, and stroke); the digestive system (hepatitis, hepatocirrhosis, cholecystitis, irritable colitis, gastric ulcer, gastritis, gastric cancer, and constipation); the metabolic/endocrine system (thyroid dysfunction and diabetes); the musculoskeletal system (sprain, muscle rupture, cartilage rupture, ligament rupture, dislocation, fracture, arthritis, bruising, and disc herniation); the skin (psoriasis, atopic dermatitis, alopecia, and athlete’s foot), the nervous system (headache, migraine, and facial paralysis), the urinary/reproductive system (urinary stones, glomerulonephritis, nephrotic syndrome, and renal tuberculosis); sexually transmitted diseases (gonorrhea, syphilis, genital herpes, and AIDS); oral diseases (cavities, periodontal disease, and wisdom teeth); accidents and addictions (burns, transport accidents, firearm accidents, cuts, stings, biting, and poisoning); and other diseases. We set the type of disease experience to 1 for each category to create a variable for the number of disease experiences by disease category. Therefore, we classified 1, 2, 3, 4, and ≥ 5 diseases within the previous year.

Furthermore, several covariates including sociodemographic, military-related characteristics, and health-related characteristics were assessed. The sociodemographic characteristics included sex (man and woman); marital status (married, once married [divorced, separated, bereavement], and unmarried); and educational level (high school or less, college or more). Military-related characteristics included military type (Army, Navy/Marine, Air Force); branch (combat, technique/administration, and special); rank (warrant officer and commissioned officer [w1-o6], non-commissioned officer [e5-e9], and enlisted soldier [e1–e4]); working area (ground operations command [GOC, eastern and western part],^30^ capital area and all military headquarters, and second operational command [SOC]); service classification (long-term and short-term military services); working type (day work and shift work); and working time (≤48, ≥49 [h/week]). Health-related characteristics included subjective health status (good and bad); physical activity (0, 1–2, and ≥3); and smoking (current, former, and non). Additional characteristics included private insurance (yes and no) and satisfaction with military medical service (satisfied, neutral, and unsatisfied).

### Statistical analysis

Chi-Squared tests and logistic regression analysis were used to analyze the association between the number of annual diseases and unmet healthcare needs. The Chi-Squared test was used to examine the significant differences in unmet healthcare needs depending on the number of diseases individuals had had in the past year. Multiple logistic regression analyses were used to determine the odds ratios (ORs) and 95% confidence intervals (CIs). Subgroup analysis was performed according to the number of diseases individuals had had in the past year and unmet healthcare needs, according to working area and insurance type. Statistical analyses were performed using SAS software, version 9.4 (SAS Institute, Cary, NC, USA). A *P* value < 0.05 was considered to indicate a statistically significant result.

## Results

The study population consisted of 5162 military personnel from the ROK Armed Forces. Of the 5162 total military personnel who had experienced one or more diseases during the past year, 1302 (25.2%) experienced unmet healthcare needs. The distribution of respondents according to disease-holding number decreased as the number of diseases increased (1: 30.0%, 2: 24.6%, 3: 18.3%, 4: 14.3%, and ≥5: 12.8%). Moreover, the proportion of respondents who reported experiencing unmet healthcare needs varied according to the number of disease holders (1: 13.4%, 2: 22.9%, 3: 29.2%, 4: 34.5%, and ≥5: 41.4%). Regarding sex, women were more likely to experience unmet healthcare needs than were men (men: 24.9%, women: 33.3%) (Table I).

**Table I:** General Characteristics of Study Observations (MHS2014–2015)

Variable	Unmet Healthcare Needs						*P* Value
	Total		Yes		No		
	N	(%)	N	(%)	N	(%)	
The number of diseases individuals had in the past year							<0.0001
1	1550	30.0	208	13.4	1342	86.6	
2	1268	24.6	290	22.9	978	77.1	
3	946	18.3	276	29.2	670	70.8	
4	736	14.3	254	34.5	482	65.5	
≥5	662	12.8	274	41.4	388	58.6	
Sex							0.0044
Man	4940	95.7	1228	24.9	3712	75.1	
Woman	222	4.3	74	33.3	148	66.7	
Marital status							0.0092
Married	940	18.2	202	21.5	738	78.5	
Single (divorced, separated, bereavement)	150	2.9	44	29.3	106	70.7	
Never married	4072	78.9	1056	25.9	3016	74.1	
Educational level							0.1274
High school or less	986	19.1	230	23.3	756	76.7	
College or more	4176	80.9	1072	25.7	3104	74.3	
Military type							<0.0001
Army	3100	60.1	860	27.7	2240	72.3	
Navy, Marine	852	16.5	190	22.3	662	77.7	
Air Force	1210	23.4	252	20.8	958	79.2	
Branch							<0.0001
Combat	3308	64.1	914	27.6	2394	72.4	
Technique Administration	1640	31.8	364	22.2	1276	77.8	
Special	214	4.2	24	11.2	190	88.8	
Rank							<0.0001
Warrant officer and Commissioned officer (W1-O6)	866	16.8	250	28.9	616	71.1	
Non-commissioned officer (E5-E9)	1726	33.4	340	19.7	1386	80.3	
Enlisted soldier (E1-E4)	2570	49.8	712	27.7	1858	72.3	
Working area							<0.0001
GOC (Eastern part)	1512	29.3	434	28.7	1078	71.3	
GOC (Western part)	1712	33.2	450	26.3	1262	73.7	
Capital area and all military headquarters	208	4.0	46	22.1	162	77.9	
SOC	1730	33.5	372	21.5	1358	78.5	
Service classification							0.6012
Long-term military service	1576	30.5	390	24.8	1186	75.3	
Short-term military service	3586	69.5	912	25.4	2674	74.6	
Working type							<0.0001
Day work	3556	68.9	816	23.0	2740	77.1	
Shift work	1606	31.1	486	30.3	1120	69.7	
Working time (hour/week)							<0.0001
≤48	2392	46.3	520	21.7	1872	78.3	
≥49	2770	53.7	782	28.2	1988	71.8	
Subjective health status							<0.0001
Good	2512	48.7	564	22.5	1948	77.6	
Bad	2650	51.3	738	27.9	1912	72.2	
Physical activity (per week)							0.0028
0	816	15.8	202	24.8	614	75.3	
1–2	1446	28.0	320	22.1	1126	77.9	
≥3	2900	56.2	780	26.9	2120	73.1	
Smoking							0.2814
Current smoker	2786	54.0	726	26.1	2060	73.9	
Former smoker	314	6.1	80	25.5	234	74.5	
Non smoker	2062	40.0	496	24.1	1566	76.0	
Private insurance							0.4289
Yes	2998	58.1	744	24.8	2254	75.2	
No	2164	41.9	558	25.8	1606	74.2	
Satisfaction with military medical service							<0.0001
Satisfied	1074	20.8	410	38.2	664	61.8	
Neutral	1250	24.2	220	17.6	1030	82.4	
Unsatisfied	2838	55.0	672	23.7	2166	76.3	
Total	5162	100.0	1302	25.2	3860	74.8	

Logistic regression analysis assessed the relationship bet-ween unmet healthcare needs and the number of diseases individuals had had within the past year. In terms of variables of interest, when analyzed on the basis of one disease group, the probability of experiencing unmet healthcare needs in a group with more disease by type also increased (2: OR = 1.83, 95% CI: 1.50–2.24; 3: OR = 2.53, 95% CI: 2.05–3.11; 4: OR = 3.10, 95% CI = 2.49–3.85; ≥5: OR = 3.85, 95% CI = 3.08–4.81). In addition, unmet healthcare needs were analyzed to have a significant correlation with sex, branch, rank, working area, service classification, and working time (Table II).

**Table II:** Factors Associated with the Number of Disease that Individuals Had in the Past 1 year and Unmet Healthcare Needs (MHS2014–2015)

	Unmet Healthcare Needs	
Variables	OR	95% CI
The number of diseases individuals had in the past year		
1	1.00	–
2	1.83	(1.50–2.24)
3	2.53	(2.05–3.11)
4	3.10	(2.49–3.85)
≥5	3.85	(3.08–4.81)
Sex		
Man	1.00	–
Woman	2.07	(1.49–2.89)
Marital status		
Married	1.00	–
Single (divorced, separated, bereavement)	1.40	(0.93–2.11)
Never married	1.35	(1.05–1.74)
Educational level		
High school or less	1.00	–
College or more	1.16	(0.97–1.39)
Military type		
Army	1.00	–
Navy, Marine	1.11	(0.87–1.41)
Air Force	1.21	(0.95–1.54)
Branch		
Combat	2.88	(1.83–4.54)
Technique · Administration	2.39	(1.51–3.77)
Special	1.00	–
Rank		
Warrant officer and Commissioned officer (W1-O6)	1.75	(1.41–2.18)
Non-commissioned officer (E5-E9)	1.00	–
Enlisted soldier (E1-E4)	1.45	(1.15–1.82)
Working area		
GOC (Eastern part)	1.58	(1.08–2.31)
GOC (Western part)	1.52	(1.04–2.23)
Capital area and all military headquarters	1.00	–
SOC	1.39	(0.93–2.08)
Service classification		
Long-term military service	1.33	(1.04–1.70)
Short-term military service	1.00	–
Working type		
Day work	1.00	–
Shift work	1.18	(1.01–1.39)
Working time (hour/week)		
≤48	1.00	–
≥49	1.36	(1.18–1.56)
Subjective health status		
Good	1.00	–
Bad	1.20	(1.04–1.37)
Physical activity (per week)		
0	1.00	–
1–2	0.87	(0.70–1.08)
≥3	1.07	(0.88–1.30)
Smoking		
Current smoker	1.00	–
Former smoker	0.88	(0.66–1.17)
Non smoker	0.90	(0.77–1.04)
Private insurance		
Yes	1.00	–
No	1.06	(0.92–1.22)
Satisfaction with military medical service		
Satisfied	2.35	(1.91–2.88)
Neutral	1.48	(1.24–1.77)
Unsatisfied	1.00	–

In terms of unmet healthcare needs experienced according to each type of disease, the experience rates of unmet healthcare needs were 1.38, 1.19, 1.62, 1.39, 1.77, and 3.09 times higher for ophthalmology or otorhinolaryngology disease, respiratory or immune disease, digestive disease, skin disease, nervous disease, and dental disease, respectively (Table III).

**Table III:** Factors of Unmet Healthcare Needs Depending on Disease Types (MHS2014–2015)

	Unmet healthcare needs	
Variables	OR	95% CI
Types of diseases (Ref: Military personnel who recently not had a disease)		
Ophthalmology or otorhinolaryngology disease	1.38	(1.21–1.59)
Respiratory or immune disease	1.19	(1.03–1.36)
Digestive disease	1.62	(1.39–1.89)
Musculoskeletal disease	1.09	(0.96–1.25)
Skin disease	1.39	(1.21–1.59)
Nervous disease	1.77	(1.54–2.03)
Dental disease	3.09	(2.70–3.54)

Table IV illustrates the results of the subgroup analysis associating unmet healthcare needs with the number of diseases individuals had had in the past year and sex, marital status, educational level, working area, working time, and private insurance. Regarding the subgroup analysis of most areas, unmet healthcare needs increase as the number of diseases increases. In terms of working area, the unmet healthcare needs experience rates of the GOC (eastern part) and SOC were significantly higher than that of those having two or more diseases. Working time was also analyzed in terms of how it affects unmet healthcare needs, and a somewhat higher unmet healthcare need rate among those working over 49 h per week was found. In the analysis of the subgroups related to private insurance, the probability of experiencing unmet healthcare needs increased according to the number of diseases, regardless of private insurance status. However, the increase in unmet healthcare needs was smaller for the group with private insurance (Table IV).

**Table IV:** Subgroup Analysis of Diseases and Unmet Healthcare Needs Experienced by Military Personnel during the Past Year

Variable	Unmet Healthcare Needs								
	1		2		3		4		≥5
	OR	OR	95% CI	OR	95% CI	OR	95% CI	OR	95% CI
Sex									
Man	1.00	1.72	(1.40–2.12)	2.56	(2.07–3.16)	3.00	(2.40–3.75)	3.59	(2.85–4.51)
Woman	1.00	9.02	(2.24–36.27)	1.37	(0.21–8.78)	6.94	(1.48–32.67)	28.48	(5.47–148.17)
Marital status									
Married	1.00	1.39	(0.79–2.43)	2.23	(1.31–3.79)	2.33	(1.27–4.26)	4.19	(2.42–7.26)
Once married (divorced, separated, bereavement)	1.00	3.07	(0.43–21.90)	0.38	(0.06–2.62)	4.87	(0.27–88.08)	58.78	(5.66–610.49)
Unmarried	1.00	1.90	(1.52–2.37)	2.72	(2.16–3.44)	3.24	(2.55–4.13)	3.73	(2.88–4.82)
Educational level									
High school or less	1.00	2.47	(1.48–4.12)	3.14	(1.82–5.43)	5.33	(3.07–9.25)	3.11	(1.80–5.37)
College or more	1.00	1.74	(1.40–2.17)	2.45	(1.95–3.08)	2.75	(2.17–3.50)	4.04	(3.16–5.18)
Working area									
GOC (Eastern part)	1.00	2.39	(1.63–3.49)	3.38	(2.29–4.97)	3.73	(2.52–5.53)	5.02	(3.40–7.42)
GOC (Western part)	1.00	1.36	(0.96–1.91)	1.99	(1.39–2.84)	3.18	(2.22–4.57)	3.77	(2.55–5.57)
Capital area and all military Headquarters	1.00	0.45	(0.09–2.14)	1.71	(0.49–5.91)	1.04	(0.22–5.00)	4.46	(1.00–19.83)
SOC	1.00	2.56	(1.78–3.67)	3.11	(2.12–4.57)	3.64	(2.36–5.60)	5.35	(3.45–8.28)
Working time (hour/week)									
≤48	1.00	1.88	(1.38–2.56)	2.34	(1.69–3.24)	2.50	(1.79–3.50)	3.60	(2.51–5.15)
≥49	1.00	1.85	(1.41–2.43)	2.75	(2.08–3.63)	3.66	(2.72–4.93)	4.27	(3.19–5.72)
Private insurance									
Yes	1.00	1.53	(1.17–2.01)	2.19	(1.67–2.86)	2.90	(2.18–3.85)	3.31	(2.49–4.39)
No	1.00	2.40	(1.76–3.28)	3.32	(2.37–4.66)	3.65	(2.57–5.17)	5.11	(3.53–7.39)

## Discussion

Our study confirms that the more the types of diseases experienced by military service personnel during 1 year, the more are their unmet healthcare needs. In addition, we found that unmet healthcare needs could be affected by sex, branch, rank, working area, service classification, working type, and working time (Table II). There is also a higher possibility of experiencing unmet healthcare needs in relation to digestive system diseases, nervous system diseases, and dental diseases compared with the musculoskeletal, respiratory, and immune diseases frequently occurring in the military (Table III). Particularly, the subgroup analysis showed that the relationship between working area, private insurance, and unmet healthcare needs was strong. Moreover, a number of diseases/year versus response was found (Table IV).

This study provides basic data for improving the medical rights and accessibility of ROK military personnel. In the case of Korea, there are currently about 200 laws and ordinances for the private sector, but about 33 laws and ordinances related to military healthcare; there is a need for more attention and effort in improving health and medical care for military personnel.^31^

Our research has some limitations. First, the MHS asked about unmet healthcare needs and diseases experienced during only 1 year. Because we used self-report data, recall bias may have occurred. To track medical history more accurately, we need to check the medical records of patients who visited the military hospital. However, because of medical law and the Personal Information Protection Act, there is a limit to how individual medical records may be accessed. Second, this study has limitations in describing the causal relationship between the type of disease and unmet healthcare needs experienced by a soldier during a 1-year cross-sectional study in terms of “Reverse causality.” Third, it would have been more meaningful to study variables of interest by disease type. However, attributable to limitations in the data, there was a limit to the number we could analyze by disease. In addition, when analyzed by disease type, there was a contradiction between the logistic regression and the inexperienced group. Fourth, it is difficult to generalize the Korean military personnel response rates in this survey, even though our group was representative based on their military characteristics. Finally, it would have been more meaningful if we directly analyzed unmet healthcare needs in ROK military and unmet healthcare need in Korean society. However, MHS is data designed specifically for military personnel only. Therefore, we could not analyze general population as control group.

Despite these limitations, our research has many strengths. First, this study is the largest survey conducted by the ROK AFMC aimed at determining the health behaviors of military personnel, and at calculating the health statistics required for health business planning and evaluation. This questionnaire survey was conducted on a total of 10 000 military personnel for 1 year by selecting representative samples in the Army, Navy, and Air Force, in consideration of the distribution of class, sickness, and region. Therefore, this is the largest healthcare-related survey conducted among military personnel. Second, the statistical analysis based on data collected for 2 years showed that unmet healthcare needs increased according to the number of illnesses soldiers experienced per year. Third, we found that unmet healthcare needs increased significantly depending on the types of diseases held by working area. These data will serve as the basis for unmet healthcare needs management in the GOC region, where the largest number of military personnel work. Fourth, we have found that when military personnel have private insurance, they are less likely to experience unmet healthcare needs than those without private insurance. Currently, the ROK military operates soldiers health insurance system,^32^ but the result of the present study will provide a basis for the expansion of this system. Fifth, as far as we know, this study is the first to examine unmet healthcare needs in ROK military according to the type of disease. This analysis of the correlations between diseases and unmet healthcare needs, which occurs within a specific military environment, will be meaningful for use in future defense policy setting and system improvement.

Our study confirmed a significant relationship between the number of diseases by type in military personnel and unmet healthcare needs. Therefore, we recommend that defense policy makers and developers should propose guidelines for ensuring the medical rights of soldiers and for improving their healthcare access. It is also recommended that all soldiers be informed that they have the right to receive medical care, and that no soldiers should fall into medical care blind spots. In addition, more attention needs to be paid to the health of military personnel. Through this study, we propose the following to guarantee the medical rights of military personnel within the Korean government and the MND. First, develop a system to prevent unmet healthcare needs and the experiencing of diseases during military service, regardless of the disease type or severity. The effects of branch, rank, working type, and working time should also be minimized. Second, analyze the causes of increased unmet healthcare needs among female military personnel compared with male personnel and prepare countermeasures against these causes. Finally, it is necessary to improve the medical accessibility of military personnel in front areas by expanding or improving the functioning of medical offices or military hospitals in the GOC area.

## Supplementary Material

Supplemental_material_usz458
