# Supplementary material for: Annual Disease Experience by Type and Correlations with Unmet Healthcare Needs among ROK Military Personnel
Source: Mil Med. 2020 May 18;185(7-8):e944–51. doi: 10.1093/milmed/usz458 (PMC7427660; doi:10.1093/milmed/usz458)
Supplement: Supplemental_material_usz458 [file supplemental_material_usz458.docx]

| **Table S1: Outpatient care statistics of military hospitals (2015-2017)** | | | | |
| --- | --- | --- | --- | --- |
|  | | **2015** | **2016** | **2017** |
| Oral medicine | | 3,341 | 3,377 | 4,273 |
| Oral surgery | | 11,093 | 7,697 | 9,452 |
| Dental clinic | | 99,167 | 103,467 | 107,892 |
| Orthodontic | | 298 | 1,990 | 2,379 |
| Periodontology | | 3,221 | 3,702 | 4,724 |
| Infectious diseases | | 1,893 | 1,961 | 1,853 |
| Internal medicine | | 182,213 | 177,979 | 188,929 |
| Internal secretion medicine | | 5,336 | 5,645 | 6,132 |
| Rheumatology | | 2,431 | 2,291 | 3,122 |
| Gastroenterology | | 9,619 | 10,608 | 11,435 |
| Circulatory medicine | | 7,197 | 7,803 | 9,787 |
| Nephrology | | 3,395 | 3,076 | 3,217 |
| Allergy and clinical immunology | | 2,570 | 2,438 | 2,503 |
| Hemato-oncology | | 1,258 | 1,248 | 1,259 |
| Pulmonology | | 5,840 | 7,266 | 8,284 |
| Surgery | | 41,917 | 38,280 | 40,395 |
| Cardiothoracic surgery | | 6,580 | 7,242 | 7,482 |
| Neurosurgery | | 178,973 | 170,989 | 190,855 |
| Orthopedics | | 418,767 | 429,551 | 439,134 |
| Plastic surgery | | 5,841 | 6,715 | 7,424 |
| Emergency | | 60,991 | 72,133 | 78,379 |
| Anesthesia pain medicine | | 4,609 | 4,041 | 3,920 |
| Urology | | 29,031 | 28,873 | 29,159 |
| Pediatrics | | 21 | 56 | 57 |
| Neurology | | 23,041 | 26,312 | 26,948 |
| Ophthalmology | | 66,631 | 69,534 | 63,598 |
| Family medicine | | 6,542 | 4,058 | 4,246 |
| Gynecology | | 1,557 | 1,698 | 2,006 |
| Otolaryngology | | 114,717 | 112,737 | 118,605 |
| Rehabilitation medicine | | 11,666 | 13,111 | 16,351 |
| Mental health medicine | | 38,242 | 41,318 | 44,099 |
| Dermatology | | 161,239 | 170,462 | 167,189 |
| Korean medicine | | 17,728 | 19,333 | 22,234 |
| Nuclear medicine | | 1 | 1 |  |
| Pathology | | 21 | 14 | 10 |
| Radiology | | 619 | 501 | 601 |
| Preventive medicine | | 24 | 13 | 843 |
| Submerge medicine | | 62 | 13 |  |
| Occupation environment medicine | | 188 | 265 | 398 |
| Laboratory medicine | | 301 | 391 | 721 |
| Source : ROK Armed Forces Medical Command (AFMC) | | |  |  |
| Unit: case |  |  |  |  |
